# Physiology and Pathology of the Brain and Nervous System

**Published:** 1869-09

**Authors:** 


### Physiology and Pathology of the Brain and Nervous System.

*Velocity of Cerebral Functions.*—The "Archives des Sciences" for April 15th, contains a paper by Dr. Adolph Hirsch, on M. F. C. Donders's experiments to determine "the velocity of the psychical functions of the brain," as detailed in the "Archives de Reichert et du Bois-Raymond," and we extract from it the following passages:

"We know now that the brain requires about fifty-thousandths of a second to distinguish and signalize the distinction between two colors, and only fifty-thousandths of a second to distinguish between two vowels which are pronounced. What is more, M. Donders has succeeded in separating these two psychical acts into their components, and he has found that the brain employs about 1-25th of a second to recognize an impression, and 1-28th of a second for an act of volition to signalize that the impression has been received. With regard to the rapidity of perception in cases of hearing, sight, and touch, and the duration of the functions of the cerebral organ, M. Hirsch observes: 'I endeavored to reply to the first of these questions in 1861, and the results which I obtained for the physiological times of the different sensations have been since confirmed by eminent physiologists, among them M. Donders, who gives as the mean of his experiments for touch, 1-7th of a second; for hearing, 1-6th; and for seeing, 1-5th. But this physiological time, as I have named the interval between the excitation, and the signal given by the manifestation of perception, comprehends a greater number—M. Donders has enumerated not less than a dozen—of acts, and divers functions of the senses, the peripheral ganglia, the nerves, brain, muscles, etc., almost all have to be accomplished in this small fraction of a second. It was important to separate as far as possible these different acts, and especially to fix the time employed in the functions of the brain, for which only a maximum limit of 1-10th of a second was known, obtained by deducting from the total physiological time the portion employed in transmission by sensor and motory nerves. But what was the minimum limit?'"

M. Donders conceived the happy thought of intercalating, in the series of functions comprised in the physiologic time, certain fresh terms of purely psychial action; and this retardation, evidently due to the intercalation of a new act of the brain, has made us acquainted with the duration of the latter.

M. Hirsch thus describes M. Donders's apparatus: "The nœmatachograph is composed of a cylinder somewhat like that of the phonautograph, on which time is registered by means of a diapason making 261 vibrations in a second, and moved by electro-magnetism, on the principle proposed by Helmholtz. These vibrations can be divided into fifths, and thus thousandths of a second obtained. The time at which the action which produces a sensation occurs is registered by the machine, and likewise that of the sensation experienced by the power experimented upon.

"The mode of accomplishing this varies according to the means of excitation employed. When an inductive current is used to give a slight shock or prick to any portion of the body, or to light up suddenly different letters, or when the spark is observed through colored glasses to produce the sensation of different colors, the current itself makes its own registry by a spark passing between the style of the diapason and the cylinder, through a sheet of blackened paper, in which it makes a little hole. The observer registers his perception by touching a key, which causes a style to mark the cylinder. To avoid the error introduced by the variable time taken by electro-magnets in attracting their armature, M. Donders prefers a purely mechanical signal. The person under observation turns aside a horizontal bar of wood carrying a point, which marks the cylinder. By holding this indicator between two fingers, and turning it right or left, two signals can be given to express different sensations.

"In experiments on hearing, the sound produced by a spring striking a pin springing from the cylinder, or by a diaapson put into sudden motion, or by the human voice, is registered by the phonautograph, or by a modification of Konig's stethescope, over which an elastic membrane is stretched communicating by two caoutchouc tubes, with two embouchures. One of these serves to transmit the sound which is to be perceived, and by the other the patient reproduces the sound he hears, so that the phonautograph registers both at the same time below the chronoscopic line of the diapason. Acting alternately upon the same excitation, by the hand and the voice, we can determine and eliminate the difference of time in the two kinds of signalling."

The above descriptions are not very clear; but, in default of better, we lay them before our readers. M. Hirsch continues his paper by describing M. Donders's experiments:

"It was first desired to find the time necessary for discriminating between two sensations of touch, and to express the distinction by different signals. To accomplish this, two similar electrodes were placed on the feet of the patient, and, by means of Pohl's permutator, a slight electric shock was given to the right foot or the left, and the patient signalled his perception by the hand on the same side. The experiments were made under two conditions—either the patient knew beforehand which foot would be operated upon, so that he could give the signal without waiting for reflection, or he did not know on which side the shock would come. In the latter case the physiological time was prolonged to the extent of one-fifteenth of a second, and that is evidently the time necessary for an observer to take notice of the side on which he was struck, and, to coordinate with this idea the act of volition, to give the signal with the corresponding hand.

"In a similar manner **M.** Donders added to the sensation of sight an alternative of perception and volition, by asking the patient to make his signal with the right hand when he perceived an object suddenly illuminated with red light, and with the left hand when it was lit up with white light. In this case the psychical action prolonged the physiological time occupied in the perception of light 0.154s. A similar result was obtained when the person experimented upon pronounced a letter suddenly exhibited to him. If the alternative was only between two letters—*a* and *i*, for example—the time occupied by the psychical act was 0.166s., calculated as a mean, and 0.124s., reckoned by the minima. When one out of five vowels had to be distinguished, the time was longer: 0.170s. in the mean, and 0.163s. if the minima were taken.

"Analogous experiments were made with hearing, by uttering a vowel sound, which the patient repeated as soon as he heard it. Sometimes the vowel to be employed was made known beforehand, and at others not. When a distinction had to be noticed between two vowels, the psychical time was 0.056s. reckoned as a mean, and 0.062s. reckoned by minima; and, when five vowels were employed, the time was 0.086s. according to means, and 0.067s. according to minima."

In experiments with two colors, the discriminating signals were made with the hand, and by the voice with the vowel sounds; this caused the times in the former case to be a little longer than in the latter. M. Donders found that when the signal consisted in pronouncing the single vowel *i*, the time was less than when *pi, ti, ki*, had to be said. The retardation caused by *p* was 0.011s., by *t* 0.022s., and by *k* 0.021s.

It was found that the sense of sight required nearly three times as long to distinguish between two letters as the ear required to distinguish between two vowel-sounds. "M. Donders tried with success to separate the time required to distinguish an alternation from that employed in a corresponding act of volition. He arranged his experiment so that the patient was only required to act on the perception of one out of several vowels which he might hear—the *i*, for example, to the neglect of the rest. In this case, attention is concentrated upon the perception of *i*. The mouth and all the respiratory apparatus were prepared to pronounc *i*, and the patient had only to give breath to it. No act of volition intervened in this case;* all that was intercalated was the act of distinguishing *i* among all the vowels which might be pronounced. In these experi-

* The patient has already willed to give the signal, and only waited for the event.

ments the psychical time was found shorter than when two functions of the brain were interposed."

In experiments upon himself, M. Donders found an act of volition to require one-twenty-eighth of a second, and one of simple distinction one-twenty-fifth.

"M. Donders made similar experiments by employing letters seen and not heard; and he found that, to distinguish a letter seen —one out of several not known in advance—does not require, sensibly, any more time than in the case of a sound heard; while it will be remembered that the entire psychical time is much greater in the former case. He explains this greater rapidity, under the circumstances specified, by remarking that the patient, having only to act on perception of the letter *i*, had its image already present in his mind."

M. Donders is now at work with another instrument—his "nœmatachometer." In this apparatus, a piece of iron is suspended by a thread, and falls when the thread is burnt, interrupting an electric current, and producing a spark which can be seen through a hole. The interval between the spark and the shock produced by the falling iron can be determined and regulated, and by means of the instrument the time can be ascertained which the mind or brain requires to decide which occurs first.—*The Student of Science, Literature, and Art.—Journal of Psychological Medicine.*

